# Phakic Posterior Chamber Intraocular Lens with a Central Hole in Treating Patients with Moderate to High Myopia: A Meta-Analysis

**DOI:** 10.1155/2019/9496326

**Published:** 2019-10-30

**Authors:** Ying Tang, Jian Ye

**Affiliations:** Department of Ophthalmology, The Daping Hospital and Research Institute of Surgery of Army Medical University (Third Military Medical University), Chongqing 400042, China

## Abstract

**Purpose:**

To evaluate the efficacy of phakic posterior chamber intraocular lens with a central hole (ICL V4c) in treating patients with moderate to high myopia.

**Methods:**

PubMed, Embase, Cochrane Library, Clinical Trial, China Biomedical Literature Database (CBM), China National Knowledge Infrastructure (CNKI), and China Science Periodical Database (CSPD) were searched online. The search included publications from the building of the library until December 2018. All randomized controlled trials containing moderate to high myopia treated by phakic posterior chamber intraocular lens with a central hole were collected. Literature search, screening literature, data extraction, and quality evaluation were independently performed by two reviewers. Meta-analysis was performed using RevMan 5.3 software.

**Results:**

Meta-analysis results based on five randomized controlled trials showed that ICL V4c and ICL without a central hole had similar UCVA results (SMD = 0.08, 95% CI (−0.71, 0.88), *P*=0.84), SE (SMD = −0.18, 95% CI (−0.52, 0.15), *P*=0.29), BCVA (SMD = −0.27, 95% CI (−0.93, 0.40), *P*=0.43), and IOP (SMD = 0.03, 95% CI (−0.24, 0.30), *P*=0.84), and the difference was not statistically significant. In addition, no complications that could jeopardize vision occurred.

**Conclusions:**

Implanting ICL V4c in patients with moderate to high myopia is safe and effective.

## 1. Background

At present, the incidence of high myopia is increasing year by year. Patients with high myopia have much stronger desires to remove their glasses than those with low to moderate myopia as a result of retinal imaging reduction, aesthetics, and other factors [1, 2]. Therefore, actively seeking a safe and effective treatment to improve the quality of life for myopia patients has become a hot topic of research [3]. Refractive surgery methods for correcting myopia mainly include corneal refractive surgery and intraocular refractive surgery. For the surgical treatment of patients with moderate to high myopia, corneal refractive surgery, such as excimer laser and femtosecond laser, has certain limitations [4, 5]. Corneal refractive surgery has a large depth of corneal ablation, which changes the biomechanics of the cornea, while intraocular refractive surgery is a phakic posterior chamber intraocular lens (ICL) implantation technique that does not require cutting the cornea, which is relatively safer [6].

The phakic posterior chamber ICL (V4c) is a foldable posterior chamber intraocular lens that can correct ametropia in the ciliary sulcus between the iris and the lens [7]. ICL implantation is highly predictable and stable because it retains the patient's natural lens regulation and can be surgically removed after implantation [8]. The design of ICL has been repeatedly enhanced and developed to improve postoperative visual quality and reduce the incidence of complications, such as pupillary block and crystalline lens opacity [9]. The central hole type ICL (ICL V4c) is a central perforated posterior chamber intraocular lens. This design increases the aqueous circulation in the eye. Compared with the previous ICL, there is no need for preoperative laser iridotomy or intraoperative iridectomy, which can reduce the pain and discomfort caused by laser iris perforation and iris hemorrhage, and can also promote the natural circulation of aqueous humor, thereby nourishing the lens itself [10, 11]. At the same time, as the diameter of the central hole of the ICL V4c lens can just allow visible light to enter, there is no optical interference and scattering phenomena, and postoperative glare and visual distortion are avoided. In all, 94.33% of patients will not notice the presence of the central hole, and it will not produce corresponding visual impairments [12].

At present, many clinical results show that ICL V4c is safe, effective, predictable, and stable [13–15]. However, to date, there has been no systematic evaluation of the therapeutic effect and safety of the phakic posterior chamber intraocular lens with a central hole in treating patients with moderate to high myopia, so we conducted a systematic review and meta-analysis to objectively evaluate this exact issue.

## 2. Materials and Methods

This review was conducted in accordance with the Preferred Reporting Items for Systematic Reviews and Meta-Analyses (PRISMA) guidelines and the recommendations of the Cochrane Collaboration.

### 2.1. Literature Search

**Figure 1:**
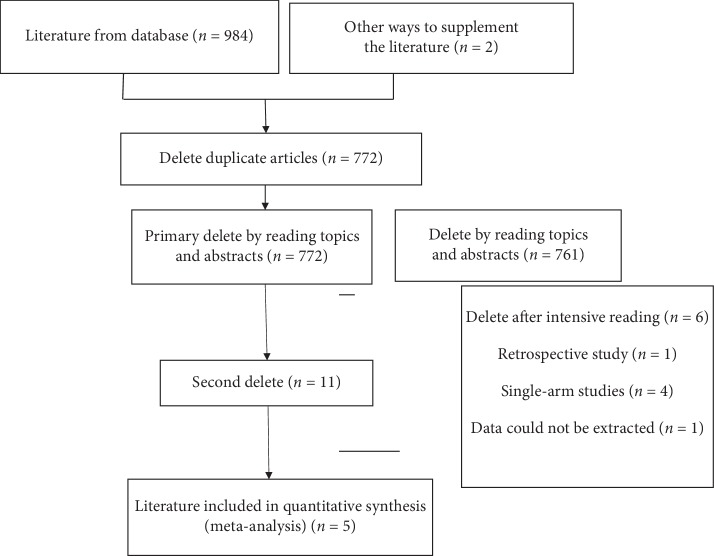
Flowchart of selection of studies for inclusion in meta-analysis.

PubMed, Embase, Cochrane Library, Clinical Trial, China Biomedical Literature Database (CBM), China National Knowledge Infrastructure (CNKI), and China Science Periodical Database (CSPD) were searched using keywords such as ICL V4c, Implantable Collamer Lens, ICL, Hole, Myopia, Myopias, Nearsightedness, etc. The combination of free words and subject words were used when searching. The search includes publications from the building of the library until December 2018. All randomized controlled trials of moderate to high myopia treated by phakic posterior chamber intraocular lens with a central hole were collected (Figure 1).

### 2.2. Eligibility Criteria

(1) Types of studies: randomized controlled trials (RCTs); language was limited to English. (2) Types of participants: patients diagnosed with moderate to high myopia, receiving posterior chamber intraocular lens, excluding medical histories of cataract, glaucoma, amblyopia, retinal detachment, diabetic retinopathy, macular degeneration, optic nerve disease, or eye inflammation. (3) Intervention: the test groups were implanted with ICL V4c, and the control groups were implanted with ICL without the central hole. (4) Outcomes: UCVA, SE, BCVA, intraocular pressure, and adverse reactions were compared.

### 2.3. Exclusion Criteria

(1) Review, conference summary, commentary, and other articles that were not treatises; (2) data loss, which led to the impossibility of trying to extract the effect amounts that could be meta-analyzed from the author's fruitless research.

### 2.4. Data Extraction

Data were extracted by two researchers using standardized electronic data extraction tables. The extracted content included the following: (1) general study characteristics, including author, country, publication time, sample size, and follow-up time; (2) general characteristics of the studies included population, age, SE, and UDVA; and (3) inconsistent content and disagreements in the process of extracting data were resolved through discussions with a third researcher.

### 2.5. Quality Assessment

The methodological quality of the included studies was independently assessed by two evaluators based on the bias risk assessment criteria recommended by the Cochrane Collaboration. The evaluation content included the following: (1) generation of random sequence; (2) allocation concealment; (3) blind method implementations; (4) incomplete report data; (5) selective report data; and (6) other bias sources. Each criterion was evaluated as “low risk bias,” “unclear,” or “high risk bias.” If the evaluation results fully met the above criteria, the probability of occurrence of bias was the smallest, and the quality level was “A”; those that partially met the above criteria indicated that the possibility of bias was moderate, and the quality level was “B”; and those that did not meet the above criteria at all are the most likely to be biased and had a quality rating of “C.” When there were differences in the evaluation results, they were discussed by the 2 initial evaluators or by the third evaluator.

### 2.6. Statistical Analyses

All combined analyses were performed by using RevMan 5.3 software. The continuous variables used standardized mean difference (SMD) and its 95% confidence interval (CI) as the statistical analysis; the heterogeneity between the studies was explored by a *Q* test and an *I*^2^ test. When *P* < 0.01 or *I*^2^ > 50%, the studies were considered heterogeneous, and the random effect model was used for analysis; otherwise, the fixed effects model was used for analysis. All the combined results were statistically significant at *P* < 0.05.

## 3. Results

### 3.1. Search Results

A total of 986 articles were collected through the related database searches and through other ways to supplement the collected literature. Import Endnote was used to delete 214 duplicates. A total of 761 articles were excluded after reading the topics and abstracts (Figure 1). Six were excluded after intensive reading of the full text. A total of 5 articles [14, 16–19] were included in the meta-analysis (Table 1).

### 3.2. Study Characteristics

All the included studies were RCTs, and the basic characteristics of the literature are shown in Table 1. The sample size was from 32 to 111 [17]. One study was from Japan, and 4 studies [14, 16, 18, 19] came from China. The follow-up period ranged from 1 month to 5 years. Four studies reported preoperative SE, and two studies did not report preoperative UDVA.

### 3.3. Methodological Quality Evaluation

One study [16] had a quality level of “A,” and the remaining studies had a quality level of “B.” The methodological quality evaluation results are shown in Table 2.

## 4. Synthesis of Results

### 4.1. Results of UCVA Meta-Analysis

**Figure 2:**
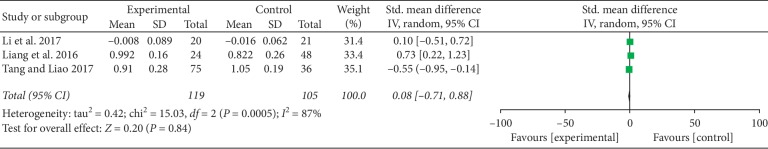
UCVA forest map after follow-up.

Three studies [14, 18, 19] reported UCVA after follow-up for a total of 224 eyes. The heterogeneity test results from the random effects model showed that *I*^2^ = 87% and *P*=0.0005. Meta-analysis results showed that SMD = 0.08, 95% CI (−0.71, 0.88), and *P*=0.84, for ICL V4c implantation and ICL without the central hole. Their UCVAs were similar after follow-up, and the difference was not statistically significant (Figure 2).

### 4.2. Results of SE Meta-Analysis

**Figure 3:**
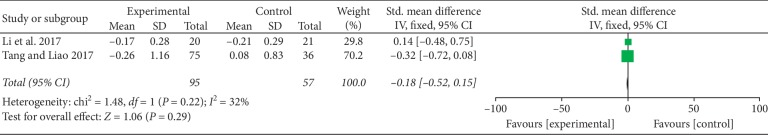
SE forest map after follow-up.

Two studies [14, 18] reported SE after follow-up for a total of 152 eyes. The heterogeneity test results showed that *I*^2^ = 32% and *P*=0.22, using a fixed effects model. Meta-analysis results showed that SMD = −0.18, 95% CI (−0.52, 0.15), and *P*=0.29 for ICL V4c implantation and ICL without the central hole. Their SEs were similar after follow-up, and the difference was not statistically significant (Figure 3).

### 4.3. Results of BCVA Meta-Analysis

**Figure 4:**
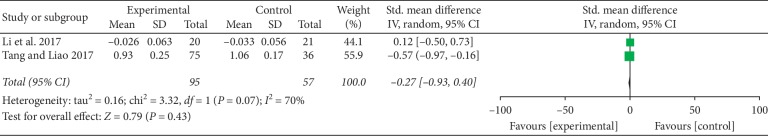
BCVA forest map after follow-up.

Two studies [14, 18] reported BCVA after follow-up for a total of 152 eyes. The heterogeneity test results showed that *I*^2^ = 70%, *P*=0.07, using a random effects model. Meta-analysis results showed that SMD = −0.27, 95% CI (−0.93, 0.40), *P*=0.43, for ICL V4c implantation and ICL without the central hole. Their BCVAs were similar after follow-up, and the difference was not statistically significant (Figure 4).

### 4.4. Results of IOP Meta-Analysis

**Figure 5:**
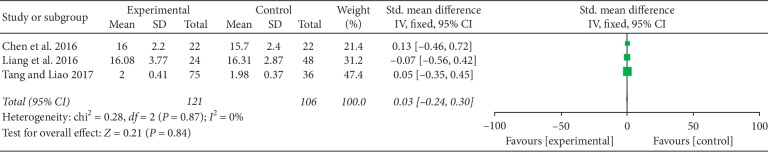
IOP forest map after follow-up.

Three studies [14, 16, 18] reported IOP after follow-up for a total of 227 eyes. The heterogeneity test results showed that *I*^2^ = 0% and *P*=0.87, using a fixed effects model. Meta-analysis results showed that SMD = 0.03, 95% CI (−0.24, 0.30), and *P*=0.84, for ICL V4c implantation and ICL without the central hole. Their IOPs were similar after follow-up, and the difference was not statistically significant (Figure 5).

### 4.5. Adverse Events

In one study [14], due to the residual viscoelastic agent used during the operation, 4 eyes had acute pupillary block in the early postoperative period, and the intraocular pressure returned to normal after a small amount of discharge with 6-point puncture drainage.

Therefore, it is important to select a viscoelastic agent that is suitable for surgery and that is easily removed during surgery without causing intraocular toxicity. During the follow-up period, no complications, such as endophthalmitis, anterior lens opacity, glaucoma, or TICL deviation from the astigmatism axis, occurred in any of the eyes. For other studies during the follow-up period, no cataract formation, pigmentation syndrome, axial rotation, glaucoma, pupillary block, or other complications that could jeopardize vision were observed.

## 5. Discussion

At present, the central hole-type ICL V4c is used in most countries and regions in the world, but a few countries and regions continue to use the nonporous ICL V4. In 2014, the central hole ICL V4c passed the Chinese SFDA; its unique 360 *μ*m central hole design has historical significance and its value is exhibited in the following points: (1) it is not necessary to cut the hole around the iris, which avoids postoperative discomfort caused by a double pupil, and this reduces tissue damage while hardly affecting the structure of the eye; (2) re-establishment of the aqueous humor cycle, reducing the incidence of turbidity in the posterior capsule as shown with *in vitro* tests by Shimizu et al. [20], who showed that ICL V4c improves the metabolism of the lens itself and the aqueous humor on its surface, reducing the incidence of postoperative cataracts; (3) ensuring the stability of intraocular pressure, as shown by studies by Higueras-Esteban et al. [21], which reveals that there is no statistically significant difference in intraocular pressure after ICL V4c implantation compared with preoperative intraocular pressure; (4) the 360 *μ*m central hole design has little interference with visual quality, ensuring good visual effects after surgery. Liang et al. [19] implanted ICL V4 and ICL V4c in two groups of patients with high myopia. The postoperative visual quality questionnaire showed that both the ICL V4c and normal lens were satisfactory, but with regard to night glare, the ICL V4c group of patients had better vision, thus increasing patient satisfaction. Most likely, because ICL V4c implantation does not require iris laser perforation, it reduces trauma to the eye, which avoids the glare caused by iris laser drilling. Does the center hole with 360 *μ*m diameter in the ICL V4c affect vision? What is the impact on visual quality? Although some studies compared the efficacy of ICL V4 and ICL V4c implantation, the conclusions were different. Therefore, we conducted a systematic review and meta-analysis to objectively evaluate the effectiveness and safety of treatment.

In this study, we included 5 items, [14, 16–19] and randomized controlled trials were performed to objectively assess the efficacy of ICL V4c implantation versus ICL implantation without the central hole for the treatment of high myopia. Meta-analysis found no significant difference in UCVA, SE, BCVA, and IOP between the two groups. Vision is the primary indicator for evaluating postoperative visual quality. UCVA is an intuitive indicator for determining the effect of refractive error correction surgery. This study compared UCVA after ICL V4c and ICL implantation and found that both have similar abilities to correct high myopia, which is consistent with the research of most scholars [22–24]. In addition, the BCVA of the ICL V4c group also showed comparable effects to the ICL group, indicating that patients with ICL V4c implantation have a stable visual state.

In this study, ICL V4c is presented as the latest type of phakic posterior chamber intraocular lens with a central hole of 360 *μ*m in diameter, which is beneficial to the circulation of aqueous humor. Traditional ICL requires laser iridotomy before surgery. In 1 study [14], due to the residual viscoelastic agent used during the operation, 4 eyes had acute pupillary block in the early postoperative period, and the intraocular pressure returned to normal after a small amount of discharge through many 6-point piercings. We compared IOP in patients with moderate to high myopia after implantation with ICL V4c and ICL, and the difference was not statistically significant; both had good safety.

In addition, this study found that ICL V4c also has very good predictability and stability compared to ICL. The study of Tang and Liao [14] showed that the mean SEs 1 day, 1 week, 1 month, and 3 months after implantation in the ICL V4c group were −0.23 ± 1.35, −0.26 ± 1.18, −0.25 ± 1.18, and −0.26 ± 1.16, respectively. The difference between the SE 3 months after ICL V4c implantation and the SE before ICL V4c implantation was statistically significant (*P* < 0.001), but the difference was not significantly different from the ICL group. A study by Li et al. [18] showed that the SE of ICL with a hole 6 months after implantation was slightly lower than that of traditional ICL after implantation, but the difference was not statistically significant. This is consistent with studies by Shimizu et al. [20] and Kamiya et al. [25]. As the surgical incision of ICL implantation is in the periphery, the operation does not involve the cornea in the central pupil area, and the peripheral 3 mm corneal incision is of little consequence to the refractive effect, which may be related to the predictability of postoperative ICL and the stable postoperative refractive status.

Our meta-analysis has certain limitations. The sample size of these studies was small, the follow-up time was insufficient, and there was heterogeneity between the studies. This meta-analysis has found that ICL V4c has good effectiveness, stability, and safety in the treatment of moderate to high myopia.

## Figures and Tables

**Table 1:** Basic characteristics of the included literature.

Studies (author, year)	Country	Number		Age		Follow-up	Pre-SE		Pre-UDVA	
		Experimental group	Control group	Experimental group	Control group		Experimental group	Control group	Experimental group	Control group
Tang and Liao, 2017 [14]	China	75	36	26.11 ± 7.41	21.31 ± 3.58	3M	−12.78 ± 4.92	−9.99 ± 3.10	NA	NA
Li et al., 2017 [18]	China	20	21	21–43	20–41	6 M	−9.57 ± 3.12	−9.92 ± 2.54	NA	NA
Liang et al., 2016 [19]	China	24	48	18–40	20–44	1 M	NA	NA	0.07 ± 0.34	0.08 ± 0.03
Shimizu et al., 2016 [17]	Japan	14	18	31.2 ± 7.6	31.2 ± 7.6	5 Y	−7.54 ± 2.40	−7.51 ± 2.42	1.35 ± 0.23	1.34 ± 0.23
Chen et al., 2016 [16]	China	22	22	26.5 ± 5.8	26.5 ± 5.8	6 M	−9.43 ± 5.01	−9.94 ± 4.88	1.34 ± 0.22	1.35 ± 0.23

Note: SE : spherical equivalent; UDVA : uncorrected distance visual acuity; M : month; Y : year; NA: data not available.

**Table 2:** Methodological quality evaluation of including literature.

Included in research (author, year)	Random allocation	Allocation concealment	Blinding of participants and personnel	Blinding of outcome assessment	Selective reporting	Incomplete outcome data	Other bias	Evidence quality
Tang and Liao, 2017 [14]	H	U	U	U	L	L	L	B
Li et al., 2017 [18]	H	U	U	U	L	L	L	B
Liang et al., 2016 [19]	H	U	U	U	L	L	L	B
Shimizu et al., 2016 [17]	U	U	U	U	L	L	L	B
Chen et al., 2016 [16]	L	L	L	L	L	L	L	A

Notes: U, unclear risk of bias; L, low risk of bias; H, high risk of bias.

## Data Availability

The data used to support this study were from NCBI-PubMed and can be found at https://www.ncbi.nlm.nih.gov/pubmed/ and https://www.cnki.net/.
